# Infection-associated sTNFR1 elevation predicts post-transplant thrombotic microangiopathy in severe aplastic anemia

**DOI:** 10.3389/fimmu.2026.1807260

**Published:** 2026-04-27

**Authors:** Ruiqing Zhou, Xiaowei Chen, Yumiao Li, Ming Zhou, Shilin Xu, Xiangjun Liu, Wengen Chen, Yuping Zhang, Shunqing Wang, Wenjian Mo

**Affiliations:** 1 Department of Hematology, Guangzhou First People’s Hospital, Institute of Blood Transfusion and Hematology, Guangzhou Medical University, Guangzhou, China; 2 Beijing Bo Fu Rui (BFR) Gene Diagnostics Co., Ltd., Beijing, China

**Keywords:** allo-HSCT, Ba, severe aplastic anemia, sTNFR1, TA-TMA

## Abstract

**Background:**

Transplant-associated thrombotic microangiopathy (TA-TMA) is a severe and potentially fatal complication following allogeneic hematopoietic stem cell transplantation (allo-HSCT). Early identification of patients at risk is essential for timely intervention and improved outcomes. This study aimed to identify biomarkers capable of predicting the early risk of TA-TMA in patients with severe aplastic anemia (SAA) undergoing allo-HSCT.

**Methods:**

In a prospective nested case–control study of patients with SAA undergoing allo-HSCT, blood samples were collected before pretreatment, before infusion, and on days +7, +14, and +28. TA-TMA cases were matched 1:2 to controls. Biomarkers were compared, and predictive performance was assessed using Receiver operating characteristic analysis and Cox models.

**Results:**

TA-TMA was associated with markedly higher mortality (59% vs. 1.3%). Soluble tumor necrosis factor receptor 1 (sTNFR1) consistently showed the strongest predictive ability, including before pretreatment (area under the curve 0.76; 95% CI, 0.60–0.91). A cutoff of 1.93 ng/mL yielded 0.88 specificity and 0.69 sensitivity. Elevated pretreatment sTNFR1 (≥1.93 ng/mL) predicted TA-TMA (hazard ratio, 6.78; 95% CI, 2.26-20.3; p < 0.001) and death (21.44; 95% CI, 2.57-179; p = 0.005). 19 had pre-transplant infections, of whom 11 (58%) developed TA-TMA versus 6 of 32 (19%) without infection. Infected patients also showed higher pretreatment sTNFR1 levels (1.93 vs. 1.64 ng/ml, p = 0.0034).

**Conclusions:**

Pretreatment sTNFR1 is a strong early predictor of TA-TMA and mortality in patients with SAA undergoing allo-HSCT, suggesting Tumor Necrosis Factor-alpha–driven inflammation precedes clinical onset. sTNFR1 may be a useful early-warning biomarker, and infection control before transplantation may reduce risk.

## 1 Introduction

Transplant-associated thrombotic microangiopathy (TA-TMA) is a severe and potentially fatal complication of hematopoietic stem cell transplantation (HSCT), with an incidence of 20–30% and mortality as high as 80% in severe cases (1). Clinically, TA-TMA presents with anemia, thrombocytopenia, schistocytes, elevated lactate dehydrogenase (LDH), kidney injury, and sometimes neurological symptoms (2, 3).Severe aplastic anemia (SAA) patients undergoing HSCT face unique risks for TA-TMA due to pre-existing infections, prolonged cytopenias, and cyclosporine-induced renal impairment. Compared with patients with hematologic malignancies, those with SAA show a significantly higher incidence of TA-TMA, as reported in multicenter and single-center studies (4, 5).

Early diagnosis is critical to improving TA-TMA outcomes. Current therapies—such as defibrotide and complement inhibitors—are more effective when initiated early (6). Prior studies have identified biomarkers like soluble C5b-9 (sC5b-9), complement factor B Ba fragment (Ba), soluble suppression of tumorigenicity 2 (sST2), and double-stranded DNA (dsDNA) as potential predictors (7–16), but their performance in patients with SAA remains unclear. Moreover, inflammatory cytokines such as Tumor Necrosis Factor-alpha (TNF-α) may be involved in TA-TMA pathogenesis (17), yet their predictive value is not well established.

To address these gaps, we conducted a prospective study assessing the temporal dynamics of multiple biomarkers—including sC5b-9, dsDNA, Ba, C-X-C motif chemokine ligand 9 (CXCL9),soluble tumor necrosis factor receptor 1 (sTNFR1), sST2,regenerating islet-derived protein 3-alpha (REG3α),serum creatinine (Scr), and LDH—in patients with SAA undergoing HSCT. We aimed to determine whether early elevations in these markers are associated with subsequent TA-TMA development, with a particular focus on identifying predictive indicators measurable before conditioning.

## 2 Materials and methods

### 2.1 Study design and patient selection

This investigation adopted a prospective nested case-control approach. Between August 2022 and June 2023, consecutive patients diagnosed with SAA who underwent allogeneic HSCT at our center were enrolled in the study. Peripheral blood samples were collected at five time points: before pretreatment, one day before cell infusion (-1 Day), and on Days +7, +14, and +28 after transplantation. Samples were processed to separate serum or plasma within two hours and stored at –80 °C.

All patients who developed TA-TMA were assigned to the TA-TMA group. For the non-TA-TMA group, a 1:2 nearest-neighbor propensity score matching (PSM) was performed based on key clinical variables, including patient age, human leukocyte antigen (HLA) mismatch status, and the presence of acute graft-versus-host disease (aGVHD). Archived blood samples from the matched cohorts were retrieved for biomarker analysis. Missing samples were noted at the following time points: 3 at before pretreatment, 1 on day +7, 2 on day +14, and 6 on day +28 (see **Supplementary Figure 1**).

Comparisons of biomarker levels between the TA-TMA and non-TA-TMA groups at each time point were performed. This study was approved by the Ethics Committee of Guangzhou First People’s Hospital (Approval No.: K-2022-111-03) and adhered to the principles outlined in the Declaration of Helsinki.

### 2.2 Diagnostic criteria for TA-TMA

The primary outcome was the development of TA-TMA within 100 days after HSCT. Diagnosis was based on the criteria by Jodele et al., requiring either histological evidence of microthrombi or fulfillment of at least five out of seven established clinical and laboratory indicators (18). (1) Elevated LDH above the upper limit of normal; (2) Proteinuria (random urine protein above the normal range or urine protein/creatinine ratio ≥2 mg/mg); (3) Hypertension (for patients <18 years, blood pressure exceeding the age-, sex-, and height-specific 95th percentile; for those ≥18 years, blood pressure ≥140/90 mmHg); (4) New-onset thrombocytopenia (platelet count <50×10^9^/L or ≥50% decrease from baseline); (5) New-onset anemia (hemoglobin below the lower limit of normal or increased transfusion requirement); (6) Evidence of microangiopathy (schistocytes in peripheral blood or histopathological confirmation); (7) Terminal complement activation (plasma sC5b-9 above the upper limit of the normal range).

The definitions of infection and fever, as well as the transplantation protocols, were consistent with those described in the referenced study (19).

### 2.3 Biomarker detection

The concentrations of sTNFR1, sST2, and REG3α were measured using a multiplex assay based on the Luminex platform (Human Premixed Multi-Analyte Kit, R&D Systems; Cat#: LXSAHM-05). CXCL9 was quantified using a commercial enzyme-linked immunosorbent assay (ELISA) kit (Human CXCL9 Quantikine ELISA Kit, R&D Systems; Cat#: DCX900). Serum levels of dsDNA were determined with the Quant-iT™ PicoGreen™ dsDNA Reagent and Kit (Thermo Fisher Scientific; Cat#: P7589). Complement components Ba and sC5b-9 were analyzed using the MicroVue Complement Ba Fragment EIA Kit and MicroVue sC5b-9 EIA Kit, respectively (Quidel Corporation).

### 2.4 Statistical analysis

Baseline continuous variables were described using medians and interquartile ranges (IQRs), and comparisons between groups were conducted with the Wilcoxon rank-sum test. Categorical variables were expressed as counts and proportions, with intergroup differences evaluated using either the chi-square test or Fisher’s exact test, as appropriate. Boxplots were constructed to illustrate biomarker distributions across time points between the TA-TMA and non-TA-TMA groups, and statistical significance was assessed using Wilcoxon rank-sum tests. To assess the discriminatory performance of each biomarker, receiver operating characteristic (ROC) curve analyses were performed. Correlation matrices were generated to examine the interrelationships among biomarkers at various time points. Overall survival (OS), defined as death from any cause within two years following transplantation, was estimated using the Kaplan–Meier method. Comparisons of OS, and TA-TMA incidence between groups were performed using the log-rank test. To assess the influence of high-risk biomarker profiles on the risks of TA-TMA, and mortality, Cox proportional hazards regression models were employed, incorporating cluster-robust standard errors to account for potential intragroup correlations introduced by propensity score matching. All statistical tests were two-sided; P-values < 0.05 were considered statistically significant. Analyses were performed using R software, version 4.4.1.

## 3 Results

### 3.1 Characteristics and clinical features of the study population

The baseline characteristics of patients before and after propensity score matching (PSM) are summarized in **Table 1**. A total of 96 patients with acquired SAA who underwent HSCT were included in the analysis, of whom 17 (17.71%) developed TA-TMA. The median time to TA-TMA onset was 29 days post-transplant (IQR: 22–36 days). Prior to PSM, patients who developed TA-TMA were significantly older than those who did not (median age: 37 vs. 27 years, p = 0.01). In addition, the TA-TMA group showed a significantly lower degree of donor–recipient HLA matching (p = 0.001) and a higher incidence of aGVHD (p < 0.001). Cytomegalovirus (CMV) infection was also more frequent among TA-TMA patients compared to those without TA-TMA (65% vs. 25%, p = 0.002). Moreover, the post-transplant mortality rate in the TA-TMA group was markedly elevated compared to the non-TA-TMA group (59% vs. 1.3%, p < 0.001). aGVHD was primarily treated with corticosteroids, while TA-TMA management generally involved discontinuation of calcineurin inhibitors (CNI) and substitution with basiliximab or mycophenolate mofetil (MMF); eculizumab was used in only one patient. Following PSM, no statistically significant differences were observed between the TA-TMA and non-TA-TMA groups with respect to patient age, HLA compatibility, or the incidence of aGVHD.

**Table 1:** Comparison of Baseline Characteristics Prior to and Following Propensity Score Matching.

	Before PSM			After PSM		
Characteristic	non-TA-TMA, N = 79	TA-TMA, N = 17	p-value^1^	non-TA-TMA, N = 34	TA-TMA, N = 17	p-value^1^
Age, Median (IQR)	27 (16, 36)	37 (31, 47)	**0.01**	30 (19, 43)	37 (31, 47)	0.089
Age bracket*, adult, n (%)	54 (68%)	15 (88%)	0.14	27 (79%)	15 (88%)	0.7
Sex,male, n (%)	46 (58%)	8 (47%)	0.4	20 (59%)	8 (47%)	0.4
Donor age, Median (IQR)	29 (24, 40)	33 (25, 37)	0.8	32 (24, 43)	33 (25, 37)	0.6
Donor sex, male,n (%)	55 (70%)	12 (71%)	>0.9	23 (68%)	12 (71%)	0.8
Type of Donor, n (%)			0.7			>0.9
HID	35 (44%)	7 (41%)		14 (41%)	7 (41%)	
MSD	20 (25%)	3 (18%)		7 (21%)	3 (18%)	
URD	24 (30%)	7 (41%)		13 (38%)	7 (41%)	
HLA disparity, n (%)			**0.001**			0.2
10/10	36 (46%)	2 (12%)		11 (32%)	2 (12%)	
9/10	9 (11%)	8 (47%)		9 (26%)	8 (47%)	
HID	34 (43%)	7 (41%)		14 (41%)	7 (41%)	
Graft source, n (%)			0.5			0.8
BM+PB	53 (67%)	10 (59%)		21 (62%)	10 (59%)	
PB	26 (33%)	7 (41%)		13 (38%)	7 (41%)	
Conditioning Reginem, n (%)			0.49			0.55
BU+PTCy+ATG	34 (43%)	9 (53%)		15(45%)	9(53%)	
FLU+CTX+ATG	5 (6.3%)	0 (0%)		2 (5.9%)	0 (0%)	
PTCy+ATG	40 (50.6%)	8 (47%)		17 (50%)	8 (47%)	
aGVHD prophylaxis, n (%)			0.8			0.6
CTX+CSA+MMF	48 (61%)	12 (71%)		23 (68%)	12 (71%)	
CTX+Tac+MMF	7 (8.9%)	2 (12%)		1 (2.9%)	2 (12%)	
MTX+CSA	19 (24%)	2 (12%)		6 (18%)	2 (12%)	
Other prophylaxis strategies	5 (6.3%)	1 (5.9%)		4 (12%)	1 (5.9%)	
PNH clone, n (%)	16 (20%)	4 (24%)	0.7	8 (24%)	4 (24%)	>0.9
TA-TMA time, Median (IQR)		29 (22, 36)			29 (22, 36)	
TA-TMA improvement, n (%)						
improve		9 (53%)			9 (53%)	
No improvement		8 (47%)			8 (47%)	
aGVHD, n (%)			**<0.001**			0.053
0	58 (73%)	8 (47%)		21 (62%)	8 (47%)	
I-II	17 (22%)	2 (12%)		9 (26%)	2 (12%)	
III-IV	4 (5.1%)	7 (41%)		4 (12%)	7 (41%)	
aGVHD time, Median (IQR)	26 (20, 29)	15 (15, 23)		26 (19, 29)	15 (15, 23)	
Infection, n (%)	30 (38%)	10 (59%)	0.11	17 (50%)	10 (59%)	0.6
EBV,positive, n (%)	1 (1.3%)	0 (0%)	>0.9	0 (0%)	0 (0%)	>0.9
CMV,positive, n (%)	20 (25%)	11 (65%)	**0.002**	8 (24%)	11 (65%)	**0.004**
Death, n (%)	1 (1.3%)	10 (59%)	**<0.001**	1 (2.9%)	10 (59%)	**<0.001**
Death Time, Median (IQR)	158	132 (56, 294)		158	132 (56, 294)	

1 Fisher’s exact test; Wilcoxon rank sum test; Pearson’s Chi-squared test; Wilcoxon rank sum exact test.

*Patients were categorized as adults or minors based on whether their age was greater than 18 years.

PSM, Propensity Score Matching; HID, Haploidentical donor; MSD, Matching sibling donors; URD, Unrelated donor; BM, bone marrow; PB, peripheral blood; BU, Busulfan; PTCy, Post Transplantation cyclophosphamide; ATG, antithymocyte globulin; MTX,methotrexate; Tac, tacrolimus; MMF, mycophenolate mofetil; FLU, fludarabine; CTX, cyclophosphamide; CSA, cyclosporin A; PNH, Paroxysmal nocturnal hemoglobinuria; TA-TMA, transplant-associated thrombotic microangiopathy; aGVHD, acute graft-versus-host disease; CMV, Cytomegalovirus; EBV, Epstein–Barr Virus; HLA, human Leukocyte Antigen; IQR, interquartile ranges. Bold values indicate statistical significance (P < 0.05).

### 3.2 Pre-transplant differences in sTNFR1 levels between TA-TMA and non-TA-TMA groups

**Figure 1:**
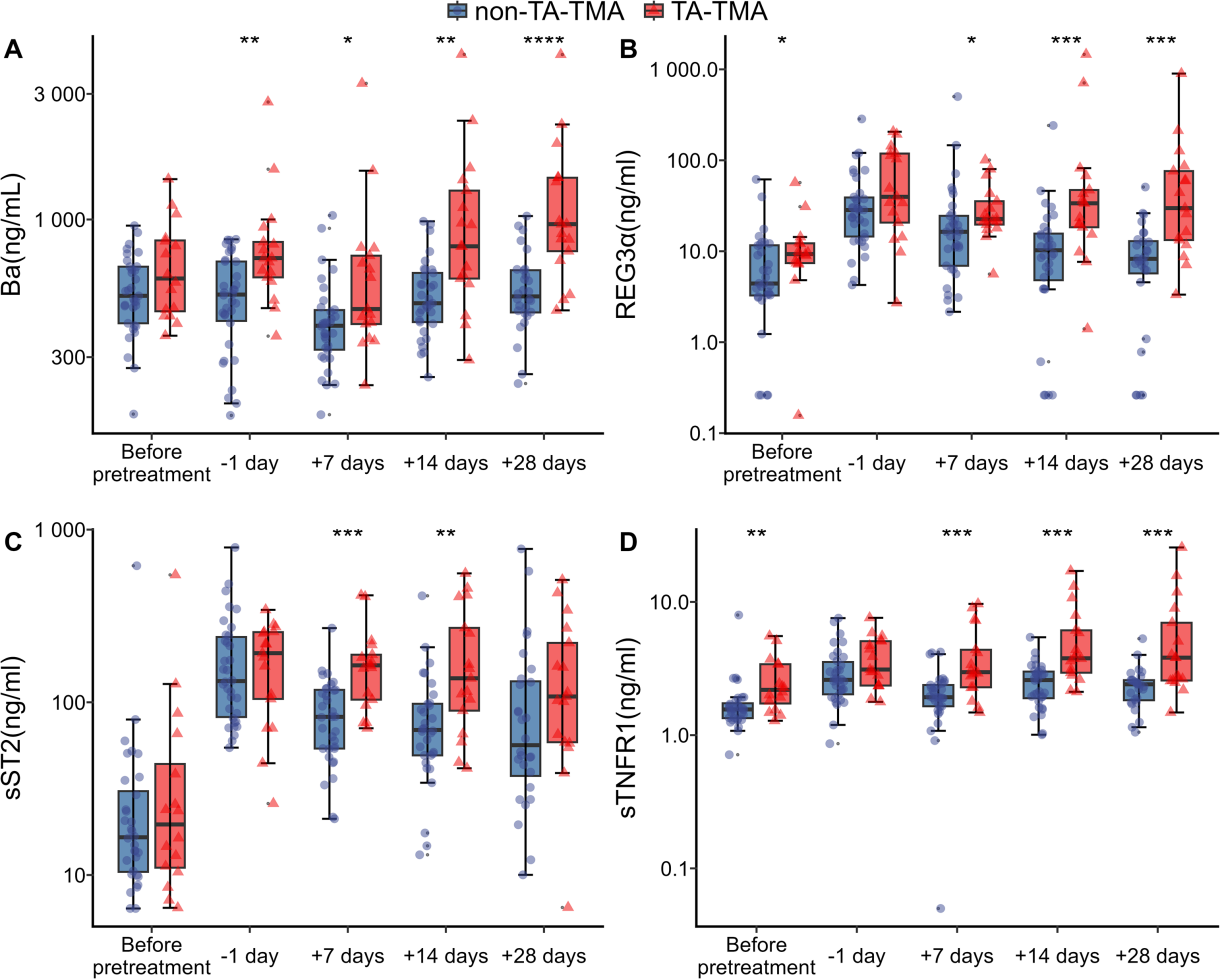
Distribution of biomarkers in non-TA-TMA (blue) and TA-TMA (red) patients measured before transplantation, -1 day and on days 7, 14, and 28 after transplantation.**(A)** Ba, **(B)** REG3α, **(C)** sST2, and **(D)** sTNFR1.The Wilcoxon rank-sum test was applied to assess differences between groups. *p < 0.05, **p < 0.01, ***p < 0.001, ****p < 0.0001. TA-TMA, transplant-associated thrombotic microangiopathy; Ba, complement factor B Ba fragment; REG3α, regenerating islet-derived protein 3-alpha; sST2, soluble suppression of tumorigenicity 2; sTNFR1, soluble tumor necrosis factor receptor 1.

**Figure 1** presents boxplots comparing biomarker distributions between patients with and without TA-TMA. Before transplantation, the TA-TMA group exhibited significantly higher levels of sTNFR1 (2.19 vs. 1.56 ng/ml, p < 0.01, **Figure 1D**) and REG3α (9.31 vs. 4.42 ng/ml, p < 0.05, **Figure 1B**) compared with the non–TA-TMA group. One day prior to cell infusion, Ba levels were significantly elevated in the TA-TMA group (713 vs. 519 ng/ml, p < 0.01, **Figure 1A**). On day 7 post-HSCT, patients with TA-TMA showed significantly higher levels of Ba (457 vs. 395 ng/ml, p < 0.05, **Figure 1A**), REG3α (23 vs. 16 ng/ml, p < 0.05, **Figure 1B**), sST2 (164 vs. 82 ng/ml, p < 0.001, **Figure 1C**), and sTNFR1 (2.97 vs. 1.93 ng/ml, p < 0.001, **Figure 1D**). On day 14 post-HSCT, these differences persisted, with elevated levels of Ba (791 vs. 481 ng/ml, p < 0.01, **Figure 1A**), REG3α (34 vs. 10 ng/ml, p < 0.001, **Figure 1B**), sST2 (138 vs. 69 ng/ml, p < 0.01, **Figure 1C**), and sTNFR1 (3.78 vs. 2.59 ng/ml, p < 0.001, **Figure 1D**) in the TA-TMA group. Because the earliest onset of TA-TMA in our cohort occurred on day 20 post-transplant, we did not assess the predictive value of biomarkers at day 28 post-HSCT. In addition, **Supplementary Figure 2** also depicts the distributions of sC5b-9, CXCL9, dsDNA, LDH, and Scr between the two groups.

**Figure 2:**
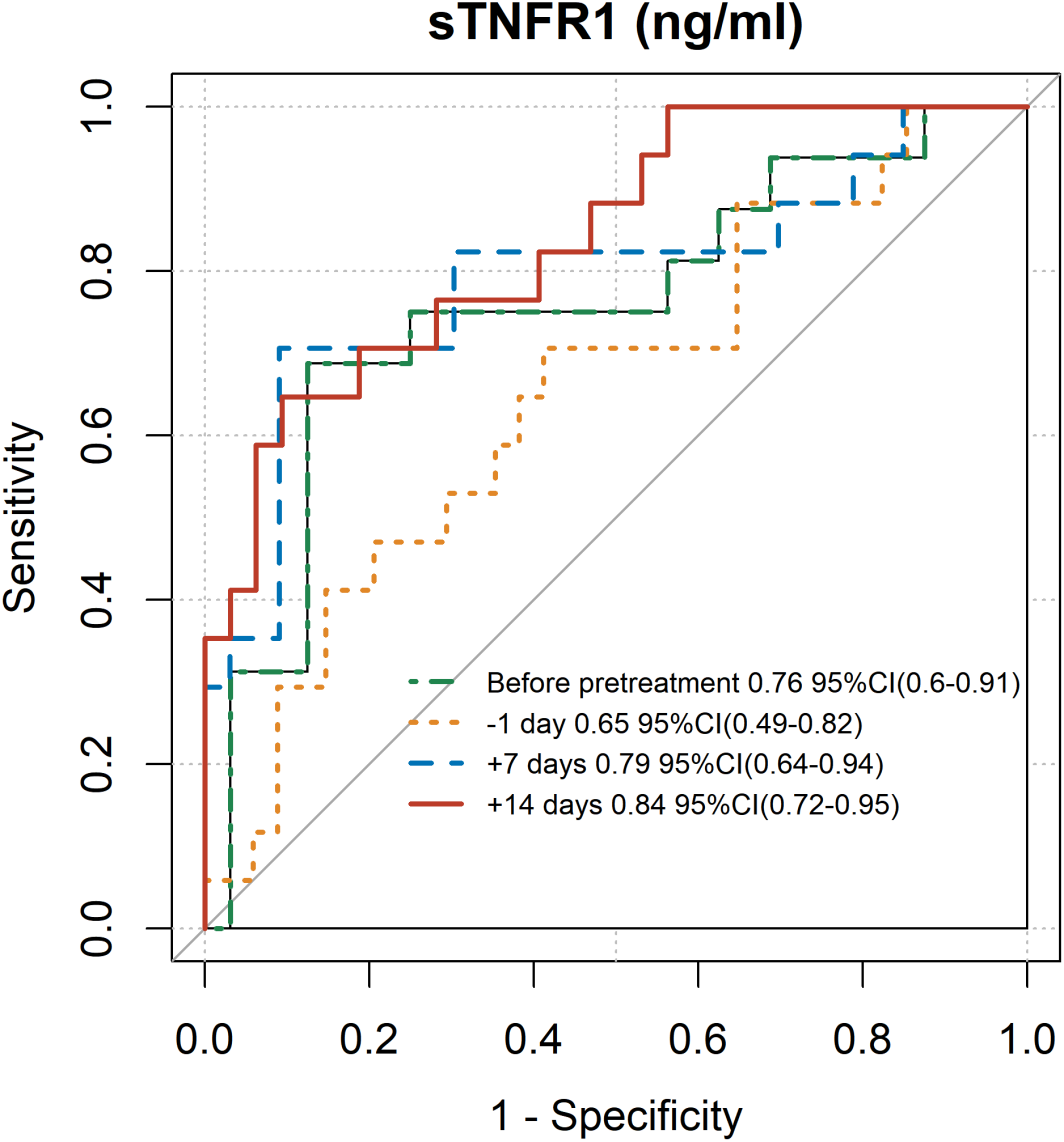
ROC curves and AUC values of sTNFR1 levels before transplantation, Day –1, Day +7, and Day +14 in discriminating TA-TMA from non–TA-TMA patients. ROC, receiver operating characteristic; AUC, area under the ROC curve; sTNFR1, soluble tumor necrosis factor receptor 1; TA-TMA, transplant-associated thrombotic microangiopathy.

Notably, sTNFR1 consistently demonstrated strong discriminatory performance between TA-TMA and non-TA-TMA patients from before transplantation through day 28 post-transplantation (**Supplementary Table 1**, **Figure 2**). The area under the ROC curve (AUC) for sTNFR1 measured before transplantation was 0.76 (95% CI, 0.60–0.91), with an optimal cut-off value of 1.93 ng/ml, achieving a specificity of 0.88 and a sensitivity of 0.69 based on the Youden index. On day 7 post-transplant, the AUC increased to 0.79 (95% CI, 0.64–0.94), and by day 14, it further improved to 0.84 (95% CI, 0.72–0.95). While Ba, REG3α, and Scr also showed some discriminatory ability before transplantation, their AUCs were consistently lower compared to sTNFR1. After transplantation, Ba, REG3α, sST2, and Scr exhibited improved predictive performance starting from day 7, whereas sC5b-9 demonstrated strong discriminative capacity only by day 28, as detailed in **Supplementary Table 1**.

### 3.3 Correlations between sTNFR1 levels before transplantation and biomarkers on days +7 and +14 post-transplantation

**Figure 3:**
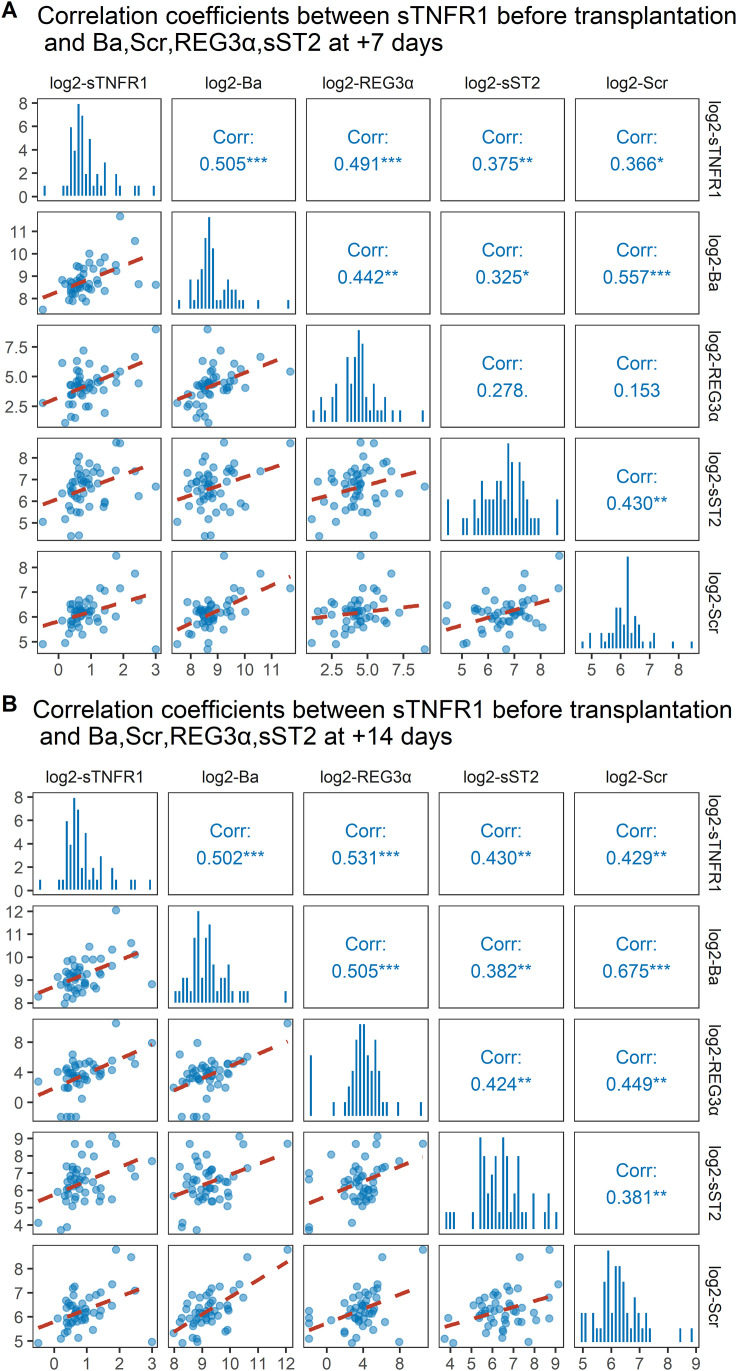
Correlation coefficients between sTNFR1 levels measured before transplantation and Ba, REG3α, sST2, and Scr biomarkers at +7 days **(A)** and +14 days **(B)** post-transplantation. All biomarker data were log_2_-transformed before analysis. Corr, correlation coefficient;p < 0.05*, p < 0.01**, p < 0.001***. sTNFR1, soluble tumor necrosis factor receptor 1; REG3α, regenerating islet-derived protein 3-alpha; sST2, soluble suppression of tumorigenicity 2; Ba, complement factor B Ba fragment; Scr, serum creatinine.

Significant correlations were identified between pre-transplant sTNFR1 levels and multiple biomarkers measured on Days +7 and +14 post-transplantation. Specifically, before-transplantation sTNFR1 was moderately correlated with Ba (r = 0.505, p < 0.001), REG3α (r = 0.491, p < 0.001), sST2 (r = 0.375, p < 0.01), and Scr (r = 0.366, p < 0.05) at day +7 (**Figure 3A**). Similar associations were observed at day +14, with correlation coefficients of 0.502 (p < 0.001) for Ba, 0.531 (p < 0.001) for REG3α, 0.430 (p < 0.01) for sST2, and 0.429 (p < 0.01) for Scr (**Figure 3B**). Additionally, strong correlations were also noted between sTNFR1 and these biomarkers when measured at the same time points on day +7 and day +14 post-transplantation (see **Supplementary Figure 3**).

### 3.4 Predictive value of sTNFR1 measured before transplantation for the cumulative incidence of TA-TMA, and OS

**Figure 4:**
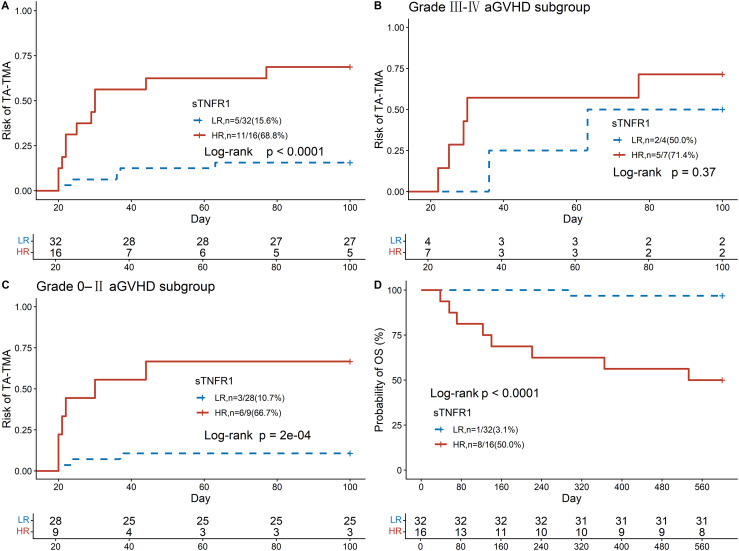
Kaplan–Meier curves comparing the LR group (sTNFR1 < 1.93 ng/ml) and HR group (sTNFR1 ≥ 1.93 ng/ml): **(A)** cumulative incidence of TA-TMA, **(B)** cumulative incidence of TA-TMA among patients with grade III–IV aGVHD, **(C)** cumulative incidence of TA-TMA among patients without aGVHD or with grade I–II aGVHD, and **(D)** overall survival probability. Group comparisons were performed using the log-rank test. sTNFR1, soluble tumor necrosis factor receptor 1; TA-TMA, transplant-associated thrombotic microangiopathy; aGVHD, acute graft-versus-host disease.

Based on sTNFR1 levels measured before transplantation, and using 1.93 ng/ml as the optimal cutoff, patients were stratified into a high-risk group (HR, sTNFR1 ≥ 1.93 ng/ml) and a low-risk group (LR, sTNFR1 < 1.93 ng/ml). Compared with the LR group, the HR group had a markedly higher risk of developing TA-TMA, with a hazard ratio of 6.78 (95% CI: 2.26–20.3, p < 0.001; **Figure 4A**), and a substantially increased risk of mortality, with a hazard ratio of 21.44 (95% CI: 2.57–179, p = 0.005; **Figure 4D**). Among patients who developed grade III–IV aGVHD, sTNFR1 levels before transplantation were not significantly predictive of TA-TMA risk (hazard ratio = 2.11, 95% CI: 0.40–11.2, p = 0.37; **Figure 4B**). In contrast, among patients without aGVHD or with only grade I–II aGVHD, sTNFR1 levels before transplantation remained a strong predictor of TA-TMA risk, with a hazard ratio of 9.06 (95% CI: 2.16–38, p = 0.003; **Figure 4C**). sTNFR1 levels before transplantation were significantly different between patients with grade III–IV aGVHD and those with grade 0–II aGVHD; however, no significant differences in sTNFR1 levels were observed between the two groups at other time points. Similarly, complement Ba and sC5b-9 levels did not differ significantly between the groups (**Supplementary Figure 4**).

After excluding three patients without available before transplantation samples, a multi-state model analysis (Transplantation → aGVHD → TA-TMA → Death) was performed in 48 patients. Nineteen developed aGVHD, of whom 8 progressed to TA-TMA, while 1 died without TA-TMA. Another 8 patients developed TA-TMA without prior aGVHD, and among the 16 patients with TA-TMA, 8 eventually died (**Supplementary Figure 5A**). Overall, 21 patients had neither TA-TMA nor aGVHD, 12 developed only aGVHD, 7 developed only TA-TMA, and 9 experienced both. Before transplantation sTNFR1 levels were significantly higher in patients with TA-TMA only (p = 0.042) and those with both aGVHD and TA-TMA (p = 0.0059) compared with patients without TA-TMA/aGVHD (**Supplementary Figure 5B**).

During the multi-state transition process, the level of sTNFR1 before transplantation significantly influenced several key transition pathways. Specifically, elevated sTNFR1 levels were associated with an increased risk of transitioning from Transplantation → aGVHD (hazard ratio = 1.66, 95% CI 1.28–2.17, p < 0.001), from Transplantation → TA-TMA (hazard ratio = 2.89, 95% CI 1.94–4.29, p < 0.0001), and from TA-TMA → Death (hazard ratio = 2.21, 95% CI 1.36–3.60, p = 0.001), as detailed in **Table 2**.

**Table 2:** Effect of sTNFR1 on transition hazards in the multi-state model.

Transition Pathways	sTNFR1 hazard ratio	95% CI	p value
Transplantation → aGVHD	1.66	1.28-2.17	<0.001
Transplantation → TA-TMA	2.89	1.94-4.29	<0.0001
aGVHD → TA-TMA	1.01	0.72-1.41	0.95
TA-TMA → Death	2.21	1.36-3.60	0.001

TA-TMA, transplant-associated thrombotic microangiopathy; aGVHD, acute graft-versus-host disease; sTNFR1, soluble tumor necrosis factor receptor 1.

### 3.5 Pre-transplant infection and the risk of TA-TMA

**Figure 5:**
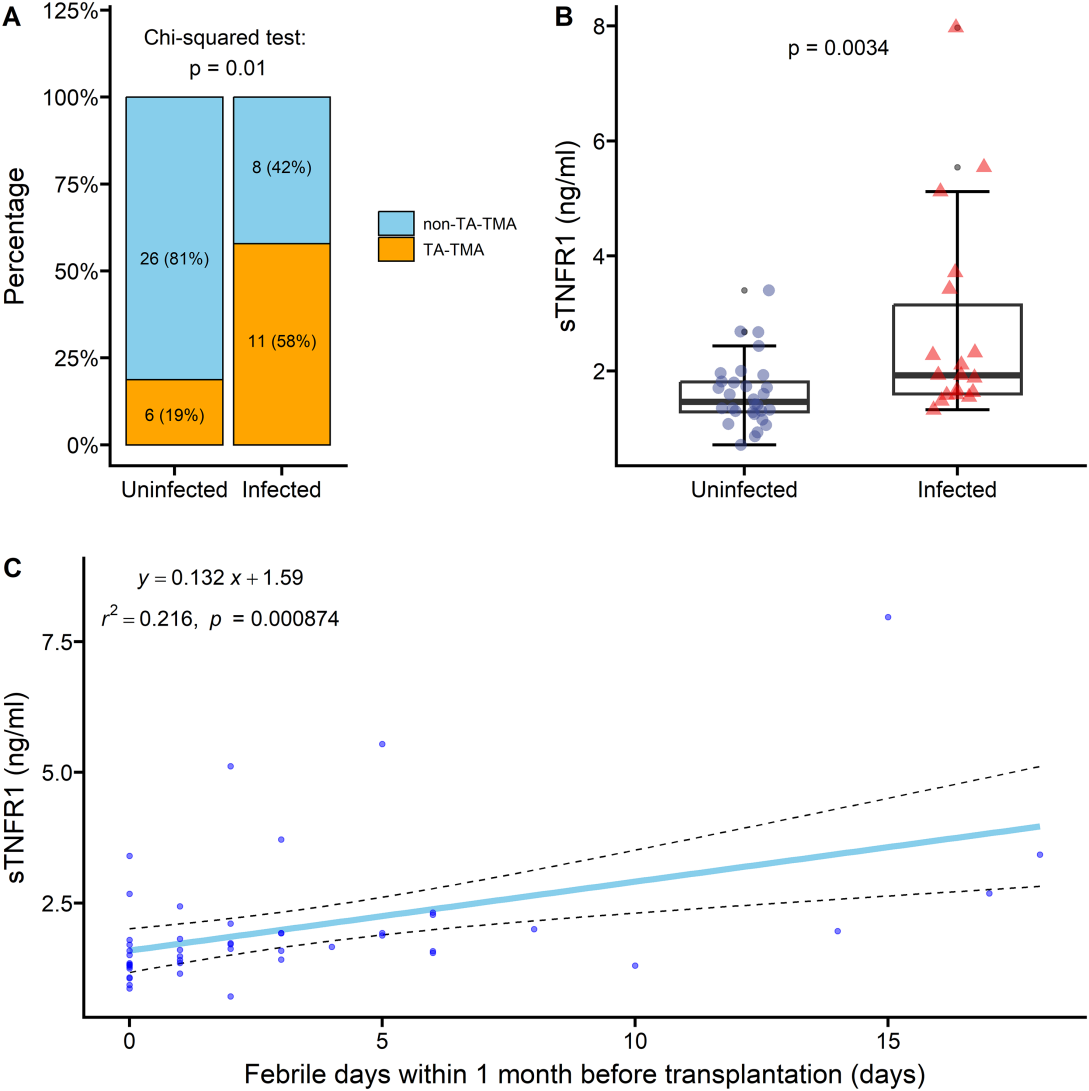
**(A)** Incidence of TA-TMA in patients with and without pre-transplant infection; **(B)** distribution of preconditioning sTNFR1 levels in patients with and without pre-transplant infection; **(C)** linear regression analysis of febrile days within 1 month before transplantation and serum sTNFR1 levels. Group comparisons were performed using the χ² test and Wilcoxon rank-sum test, as appropriate. sTNFR1, soluble tumor necrosis factor receptor 1; TA-TMA, transplant-associated thrombotic microangiopathy.

Among the 51 transplant recipients, 19 had pre-transplant infections, of whom 11 (58%) developed TA-TMA; in contrast, only 6 of the 32 patients without infection (19%) developed TA-TMA (χ² test, p = 0.01; **Figure 5A**). Among the infected patients, eleven had bacterial infections, seven had mixed bacterial and fungal infections, and one had a fungal infection. In addition, patients with pre-transplant infections had significantly higher preconditioning sTNFR1 levels compared with those without infection (1.93 vs. 1.64 ng/ml, p = 0.0034; **Figure 5B**). Furthermore, linear regression analysis showed that for each additional febrile day within 1 month before transplantation, serum sTNFR1 levels increased by an average of 0.132 ng/ml (β = 0.132, SE = 0.037, p = 0.0009). The model explained 21.6% of the variance in sTNFR1 levels (R² = 0.216, p = 0.0009; **Figure 5C**).

## 4 Discussion

In this study, we compared the differences in biomarker levels between TA-TMA and non-TA-TMA patients before and after transplantation. We found that sTNFR1 could predict the risk of TA-TMA as early as before transplantation (hazard ratio = 6.78, 95% CI 2.26–20.3, p = 0.001), preceding complement Ba and sST2 factors. Moreover, the ability of sTNFR1 to distinguish between TA-TMA and non-TA-TMA patients remained consistently high, with an AUC of 0.76 before transplantation, 0.79 at +7 days, and 0.84 at +14 days. We also observed strong correlations between sTNFR1 and complement Ba, REG3α, Scr, and sST2. Our findings, combined with previous studies (17), suggest that not only complement system activation but also inflammatory pathways—particularly those mediated by TNF-α—are involved in the pathogenesis of TA-TMA. Furthermore, the TNF-α inflammatory pathway, the complement alternative pathway, and endothelial cell injury appear to be interconnected. This may indicate that these proteins share similar patterns of change during physiological or pathological processes, or that they influence each other directly. Such correlations may provide important insights into the progression of the disease and underlying biological processes. We also observed that patients with pre-transplant infections had higher sTNFR1 levels and an increased incidence of TA-TMA. Based on these findings, we hypothesize that pre-transplant infections elevate sTNFR1 levels, which in turn may activate the complement system and induce endothelial injury, thereby increasing the risk of post-transplant TA-TMA.

There is further evidence that individuals with atypical hemolytic uremic syndrome present with markedly elevated sTNFR1 levels compared to healthy subjects, in conjunction with complement activation (both proximal and terminal), systemic inflammatory responses, endothelial dysfunction, and increased indicators of coagulation and kidney injury (20). These findings further imply that TA-TMA may share similar features, consistent with the patterns observed in our data. sTNFR1 is a soluble form of Tumor Necrosis Factor Receptor 1, which is shed from the cell membrane through cleavage or secretion processes. It acts as a natural antagonist of TNF-α, thereby reducing cytokine-induced inflammatory responses (21). Elevated levels of sTNFR1 have also been associated with the progression of kidney diseases (22, 23), and sTNFR1 has been identified as a strong prognostic marker for all-cause mortality in patients with chronic kidney disease and type 2 diabetes (24).

In our study, 11 patients (11.46%) died within two years after transplantation, among whom 10 deaths were attributed to TA-TMA and 1 death resulted from graft dysfunction. The level of sTNFR1 before transplantation could predict the risk of death (hazard ratio = 21.44, 95% CI 2.57–179, p = 0.005). Furthermore, in the multistate transition model (Transplantation → aGVHD → TA-TMA → Death), sTNFR1 measured before transplantation was also significantly associated with an increased risk of transition from TA-TMA to Death (hazard ratio = 2.21, 95% CI 1.36–3.60, p = 0.001).

Recent evidence suggests that early prophylactic administration of eicosapentaenoic acid and N-acetylcysteine may lower the incidence of TA-TMA in high-risk individuals (5). In addition, separate findings indicate that using defibrotide prophylactically during the conditioning phase and early post-transplant recovery may contribute to the prevention of TA-TMA (25). Therefore, the concentration of sTNFR1 before transplantation may serve as an indicator for early risk stratification of TA-TMA and could guide preventive interventions. Moreover, the complement inhibitor eculizumab has been reported to be effective in only about 66% of TA-TMA patients (26), leaving nearly 40% of patients with poor response to complement inhibition therapy. TNF-α could thus represent an alternative therapeutic target in these cases.

Previous studies have reported that Ba levels at +7 days post-transplantation (10) and sST2 levels at +14 days post-transplantation (12) could predict the early risk of TA-TMA. Consistent with these findings, our data also support that Ba and sST2 are early predictors of TA-TMA: Ba levels showed significant differences between TA-TMA and non-TA-TMA patients from -1 day through to +28 days post-transplantation; sST2 levels differed significantly at +7 and +14 days post-transplantation but not at +28 days.

Additionally, our data revealed that REG3α levels significantly differed between TA-TMA and non-TA-TMA patients at all evaluated time points (before transplantation, +7 days, +14 days, and +28 days post-transplantation), a finding not previously reported. REG3α is an antimicrobial peptide primarily produced by Paneth cells, playing a protective role for gastrointestinal epithelial cells against Gram-positive bacterial infections. REG3α has been associated with acute gastrointestinal GVHD, where damage to the intestinal mucosa leads to elevated serum REG3α concentrations (27). Since TA-TMA can also involve the gastrointestinal vascular system, manifesting as hemorrhagic and ischemic colitis (26, 28), TA-TMA patients may likewise exhibit elevated blood REG3α levels. Conversely, intestinal mucosal injury may itself increase the risk of TA-TMA, thus explaining the early rise in REG3α concentrations.

Although our study identified potential early predictive biomarkers for TA-TMA, several limitations should be acknowledged. First, as a single-center study with a relatively small sample size, its statistical power is limited. Second, all enrolled patients were diagnosed with SAA, which may restrict the generalizability of our findings to transplant recipients with other underlying conditions. Therefore, a multicenter prospective cohort study is warranted to further validate our results before they can be applied more broadly in clinical practice.

In summary, our results indicate that the inflammatory pathway mediated by TNF-α is activated prior to the development of TA-TMA. Higher levels of sTNFR1 measured before transplantation correlate with a greater risk of TA-TMA occurrence and mortality after transplantation. These findings hold important clinical implications, implying that sTNFR1 could be a useful biomarker for early TA-TMA prevention, while TNF-α represents a promising therapeutic target for this disease. Moreover, pre-transplant infections were associated with elevated sTNFR1 levels, indicating that effective infection control prior to transplantation may help reduce sTNFR1 levels and subsequently lower the risk of TA-TMA.

## Data Availability

The raw data supporting the conclusions of this article will be made available by the authors, without undue reservation.
