# Supplementary material for: Infection-associated sTNFR1 elevation predicts post-transplant thrombotic microangiopathy in severe aplastic anemia
Source: Front Immunol. 2026 Apr 27;17:1807260. doi: 10.3389/fimmu.2026.1807260 (PMC13158071; doi:10.3389/fimmu.2026.1807260)
Supplement: Supplementary file 1 [file Table1.docx]

**Supplementary Table 1.** AUC values, optimal cutoff values, specificity, and sensitivity of sTNFR1, Ba, REG3α, sST2, Scr,and sC5b-9 levels in distinguishing TA-TMA at before transplantation, -1 day, +7 days , +14 days, and +28 days.

| Time | Marker | AUC | 95% CI | Threshold | Specificity | Sensitivity |
| --- | --- | --- | --- | --- | --- | --- |
| Before pretreatment | Ba(ng/mL) | 0.62 | 0.44-0.8 | 783.22 | 0.91 | 0.38 |
|  | sC5b-9(ng/ml) | 0.59 | 0.41-0.76 | 132.73 | 0.48 | 0.75 |
|  | REG3α(ng/ml) | 0.69 | 0.53-0.85 | 7.19 | 0.66 | 0.88 |
|  | sST2(ng/ml) | 0.55 | 0.36-0.74 | 23.45 | 0.69 | 0.5 |
|  | sTNFR1(ng/ml) | 0.76 | 0.6-0.91 | 1.93 | 0.88 | 0.69 |
|  | Scr(umol/L) | 0.68 | 0.53-0.84 | 56.5 | 0.36 | 0.94 |
| -1 day | Ba(ng/mL) | 0.75 | 0.61-0.89 | 573.27 | 0.68 | 0.82 |
|  | sC5b-9(ng/ml) | 0.56 | 0.38-0.74 | 257.82 | 0.94 | 0.24 |
|  | REG3α(ng/ml) | 0.64 | 0.46-0.82 | 91.02 | 0.91 | 0.41 |
|  | sST2(ng/ml) | 0.53 | 0.35-0.71 | 158.54 | 0.59 | 0.65 |
|  | sTNFR1(ng/ml) | 0.65 | 0.49-0.82 | 2.74 | 0.59 | 0.71 |
|  | Scr(umol/L) | 0.73 | 0.58-0.88 | 85.5 | 0.79 | 0.59 |
| +7 days | Ba(ng/mL) | 0.7 | 0.54-0.86 | 598.87 | 0.88 | 0.47 |
|  | sC5b-9(ng/ml) | 0.6 | 0.43-0.77 | 196.7 | 0.68 | 0.59 |
|  | REG3α(ng/ml) | 0.7 | 0.55-0.85 | 17 | 0.58 | 0.88 |
|  | sST2(ng/ml) | 0.82 | 0.7-0.95 | 154.63 | 0.97 | 0.65 |
|  | sTNFR1(ng/ml) | 0.79 | 0.64-0.94 | 2.72 | 0.91 | 0.71 |
|  | Scr(umol/L) | 0.75 | 0.6-0.91 | 85.5 | 0.91 | 0.59 |
| +14 days | Ba(ng/mL) | 0.78 | 0.62-0.94 | 568.14 | 0.7 | 0.82 |
|  | sC5b-9(ng/ml) | 0.6 | 0.41-0.78 | 322.88 | 0.94 | 0.35 |
|  | REG3α(ng/ml) | 0.8 | 0.67-0.94 | 15.34 | 0.75 | 0.82 |
|  | sST2(ng/ml) | 0.75 | 0.6-0.91 | 101.66 | 0.78 | 0.71 |
|  | sTNFR1(ng/ml) | 0.84 | 0.72-0.95 | 3.49 | 0.91 | 0.65 |
|  | Scr(umol/L) | 0.77 | 0.62-0.91 | 80.5 | 0.76 | 0.71 |
| +28 days | Ba(ng/mL) | 0.85 | 0.73-0.97 | 673.12 | 0.82 | 0.82 |
|  | sC5b-9(ng/ml) | 0.85 | 0.74-0.96 | 225.87 | 0.75 | 0.88 |
|  | REG3α(ng/ml) | 0.83 | 0.69-0.96 | 25.57 | 0.93 | 0.59 |
|  | sST2(ng/ml) | 0.66 | 0.49-0.83 | 53.14 | 0.5 | 0.88 |
|  | sTNFR1(ng/ml) | 0.8 | 0.66-0.94 | 2.53 | 0.68 | 0.82 |
|  | Scr(umol/L) | 0.61 | 0.44-0.78 | 109 | 0.93 | 0.29 |

sC5b-9, soluble C5b-9; Ba, complement factor B Ba fragment; sTNFR1, soluble tumor necrosis factor receptor 1; sST2, soluble suppression of tumorigenicity 2; REG3α, regenerating islet-derived protein 3-alpha; Scr, serum creatinine; AUC, area under the curve; TA-TMA, transplant-associated thrombotic microangiopathy.


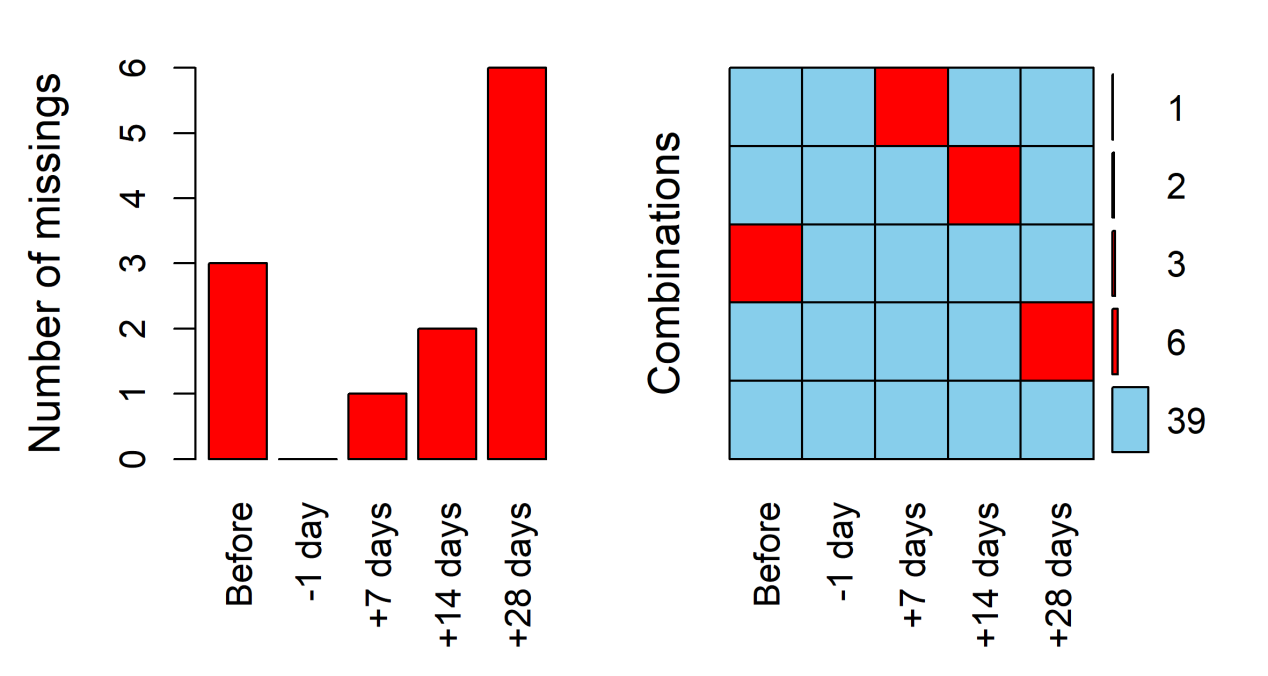


**Supplementary Figure 1**. Sample missing data at each time point.


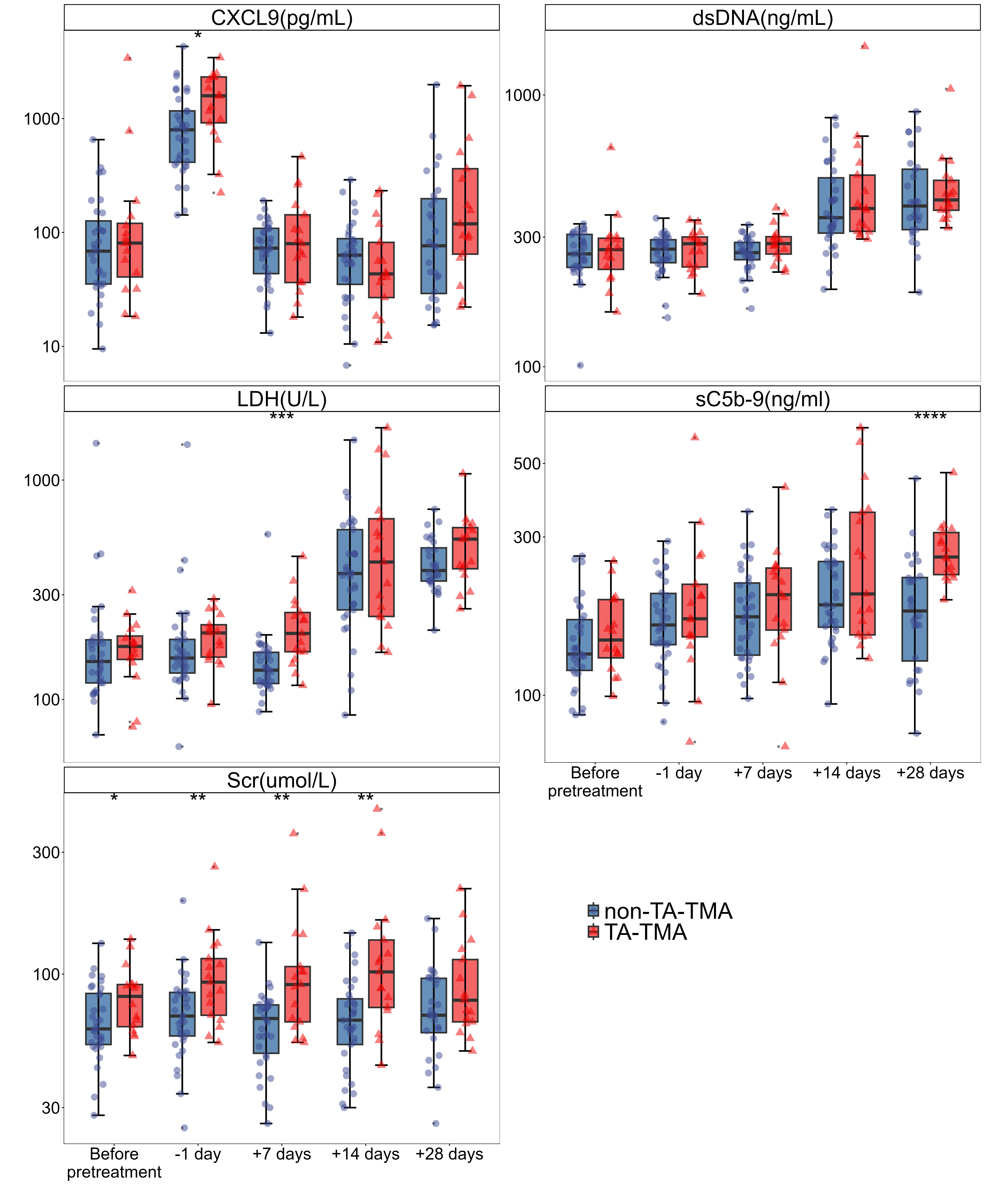


**Supplementary Figure 2**. Distribution of biomarkers (CXCL9, dsDNA, LDH, sC5b-9, and Scr) in non-TA-TMA (**blue**) and TA-TMA ((**red**) patients measured before transplantation, -1 day, and on days 7, 14, and 28 after transplantation. The Wilcoxon rank-sum test was applied to assess differences between groups.p < 0.05 *,p < 0.01 **, p < 0.001***,p < 0.0001 ****. dsDNA, double-stranded DNA; CXCL9, C-X-C motif chemokine ligand 9; LDH, lactate dehydrogenase; sC5b-9, soluble C5b-9; Scr, serum creatinine; TA-TMA, transplant-associated thrombotic microangiopathy.


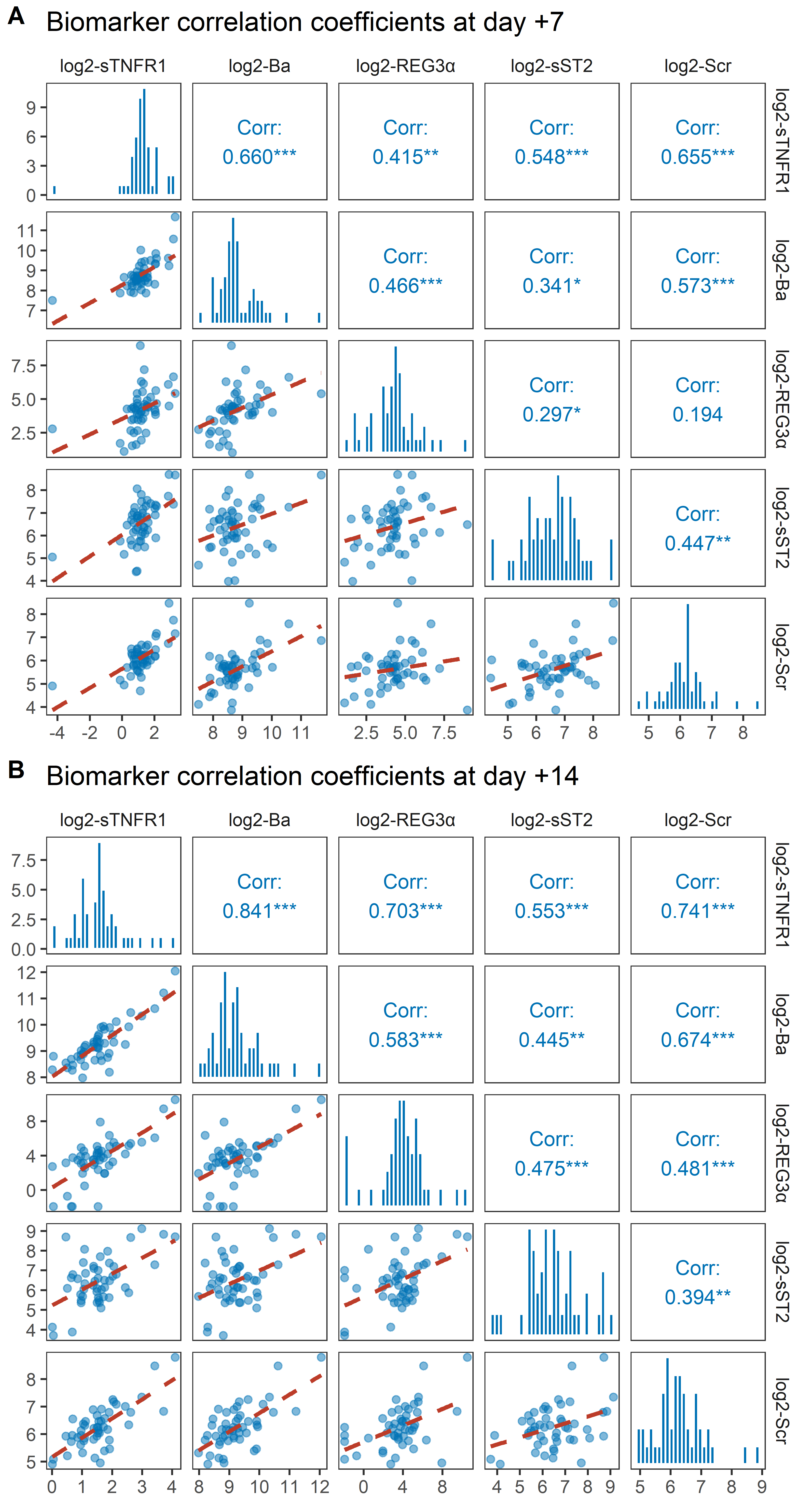


**Supplementary Figure 3**. Correlation coefficients between sTNFR1 and Ba, REG3α, sST2, Scr biomarkers at +7 days (**A**) and +14 days (**B**) after transplantation. All data were log2 transformed. Corr, correlation coefficient; p < 0.05*, p < 0.01**, p < 0.001***. Ba, complement factor B Ba fragment; sTNFR1, soluble tumor necrosis factor receptor 1; sST2, soluble suppression of tumorigenicity 2; REG3α, regenerating islet-derived protein 3-alpha; Scr, serum creatinine.


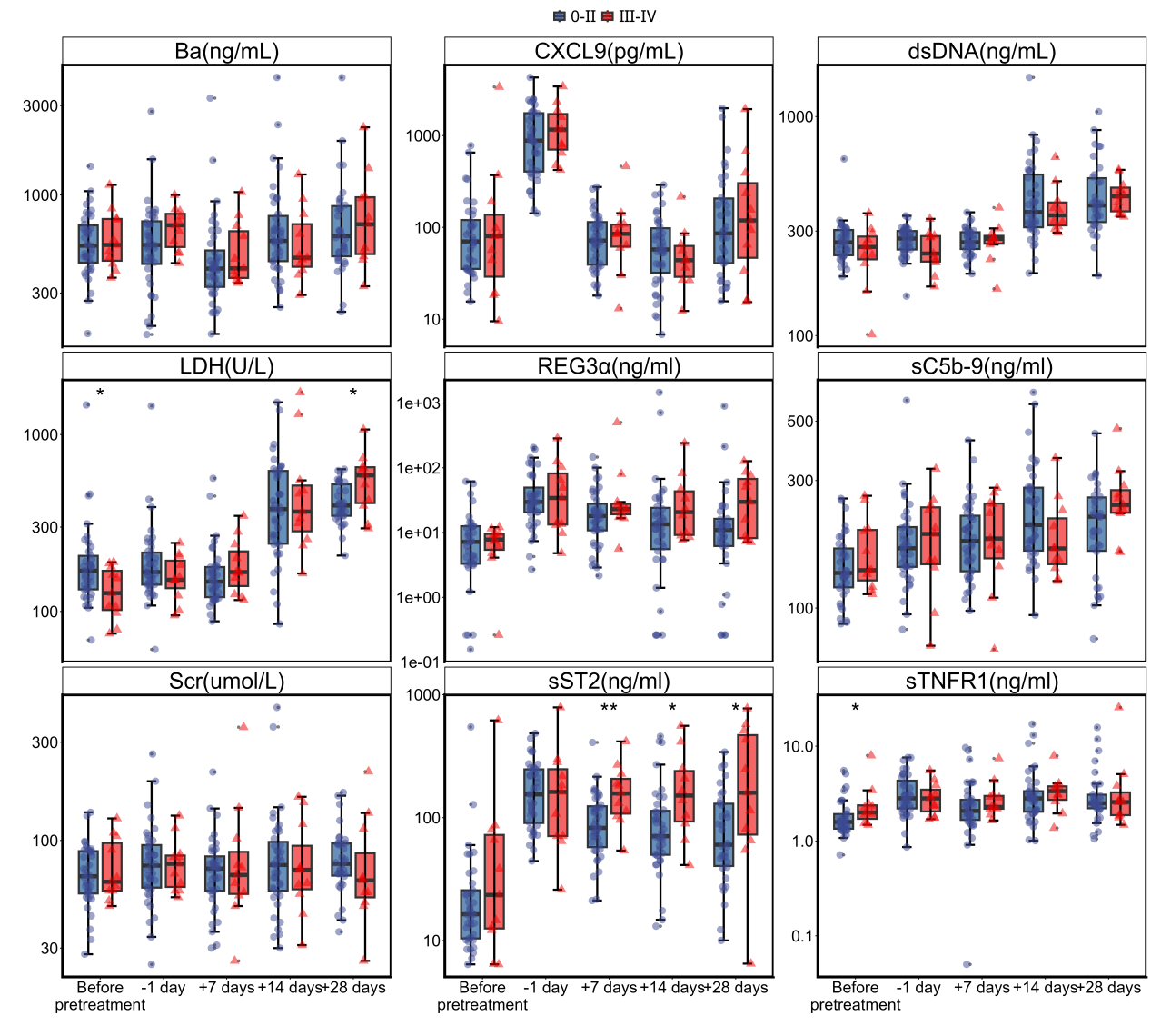


**Supplementary Figure 4**. Bar chart showing the distribution of Ba, CXCL9, dsDNA, LDH, REG3α, sC5b-9, Scr, sST2, and sTNFR1 at five time points (before transplantation,-1 day, +7 days, +14 days, and +28 days) in patients with 0-II aGVHD (**blue**) and III-IV aGVHD (**red**). The Wilcoxon rank-sum test was used to compare differences between the two groups; p < 0.05*, p < 0.01**. sC5b-9, soluble C5b-9; Ba, complement factor B Ba fragment; sTNFR1, soluble tumor necrosis factor receptor 1; sST2, soluble suppression of tumorigenicity 2; REG3α, regenerating islet-derived protein 3-alpha; Scr, serum creatinine; dsDNA, double-stranded DNA; CXCL9, C-X-C motif chemokine ligand 9; LDH, lactate dehydrogenase; aGVHD, Acute Graft-versus-Host Disease.


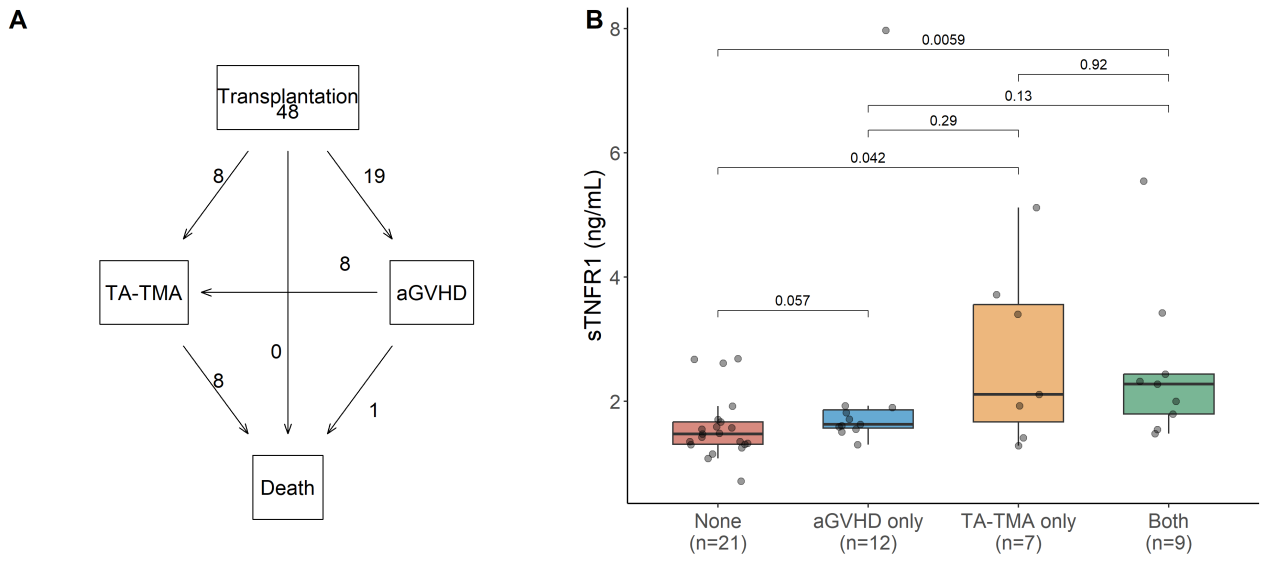


**Supplementary Figure 5**. Patient transition pathways and pre-transplantation sTNFR1 levels in relation to aGVHD and TA-TMA. **A**) Multi-state model illustrating patient transitions after transplantation (Transplantation → aGVHD → TA-TMA → Death). Numbers of patients in each transition pathway are shown. **B**) Comparison of pre-transplantation sTNFR1 levels among four groups: no aGVHD/TA-TMA (n = 21), aGVHD only (n = 12), TA-TMA only (n = 7), and both aGVHD and TA-TMA (n = 9). Significance between groups was assessed using the Wilcoxon test. aGVHD, Acute Graft-versus-Host Disease; TA-TMA, transplant-associated thrombotic microangiopathy; sTNFR1, soluble tumor necrosis factor receptor 1.
